# Therapeutic and Adverse Effect of Anti-PD1 Immunotherapy in Melanoma: A Retrospective, Single-Institute Study of 222 Patients

**DOI:** 10.3390/cancers15153966

**Published:** 2023-08-04

**Authors:** Grethe Eikenes, Gabriella Liszkay, Tímea Balatoni, Kata Czirbesz, Karen Hunyadi, Zsófia Kozéki, Mihály Tamás Kispál, Fanni Baranyai, Tímea Danyi, Katalin Bőcs, István Kenessey

**Affiliations:** 1 Department of Dermato-Oncology, National Institute of Oncology, H1122 Budapest, Hungary; grethe.eikenes@sus.no (G.E.); balatoni.timea@oncol.hu (T.B.); czirbesz.kata@oncol.hu (K.C.); hunyadi.karen@oncol.hu (K.H.); kozeki.zsofia@oncol.hu (Z.K.); kispal.mihaly@oncol.hu (M.T.K.); baranyai.fanni@oncol.hu (F.B.); danyitimi@gmail.com (T.D.); 2 National Tumor Laboratory Project, H1122 Budapest, Hungary; kenessey.istvan@oncol.hu; 3 Department of Diagnostic Radiology, National Institute of Oncology, H1122 Budapest, Hungary; bocs.katalin@oncol.hu; 4 National Cancer Registry, National Institute of Oncology, H1122 Budapest, Hungary; 5 Department of Pathology, Forensic and Insurance Medicine, Semmelweis University, H1091 Budapest, Hungary

**Keywords:** melanoma, anti-PD1, therapy, immune-related side effects, outcome

## Abstract

**Simple Summary:**

The introduction of checkpoint inhibitors, such as anti-PD1 and anti-CTLA4 approaches, resulted in a breakthrough step in the outcome of advanced melanoma. However, next to the improved efficacy, a wide range of side effects appeared. During our analysis, we explored the effects and side effects of checkpoint inhibitors among single-center enrolled patient samples with advanced (stage IV and III) melanoma. We have concluded that despite the range of immunotherapeutic options is getting wider, in the management of melanoma patients, anti-PD1 monotherapy remains an important, effective, and safe method. In addition, positive significant correlation was revealed between the immune-related side effects and therapeutic efficacy.

**Abstract:**

Background: The introduction of immuno- and targeted therapeutic modalities meant a breakthrough step in the therapy of melanoma. As a checkpoint inhibitor, the more effective and less toxic anti-PD1 therapy followed an anti-CTLA4 approach. Methods: From our patient pool, 222 advanced melanoma cases were selected, where anti-PD1 (pembrolizumab, nivolumab) therapy was initiated between March 2015 and December 2020. During our retrospective analysis, the efficacy and safety of the therapy were assessed. Results: The median follow-up was 16 months (interval: 0–64 months), and 150 patients (67.6%) received therapy in the first line, while second and third line therapy was performed among 72 patients (32.4%) for the median of 7.0 months (0–60). In 50 cases, BRAF mutations were detected. Ninety-six patients showed objective response (11.3% CR, 32.0% PR). The median PFS was 10.0 months (0–60), and the median OS was 23.0 months (0–64). Autoimmune side effects were found in 79 patients (35.5%); grade 3 occurred in 6.3% of the cases, while 1 patient died due to fulminant pneumonitis (0.25%). Conclusion: Although the range of immunotherapeutic options is getting wider, in the management of melanoma patients, anti-PD1 monotherapy remains an important, effective, and safe method. However, significant correlation was found between the immune-related side effects and therapeutic efficacy.

## 1. Introduction

Over the past decades, the incidence and mortality of cutaneous melanoma has been increasing constantly; nowadays, GLOBOCAN estimates there are approx. 320,000 new cases and 57,000 deaths worldwide annually [1,2]. In contrast to the growing global burden, survivorship of melanoma has not changed significantly for a while, and median survival of advanced-stage disease remains in the range of 6–10 months [3]. In 2011, the FDA accepted the anti-CTLA4 immuno- and BRAF inhibitory targeted therapeutic modalities, which at last resulted in a breakthrough for the treatment of melanoma patients with poor expected outcomes [4,5]. In 2014, the second introduced checkpoint inhibitory agent was anti-PD1, which reactivates the function of T-lymphocytes and proved to be more effective and less toxic compared to anti-CTLA4 medicines [6].

In Hungary, incidence rates have doubled, while mortality rates have stagnated in the last two decades, with approx. 3000 and 350 annual cases in 2019, respectively [7]. Since the Hungarian oncological protocols usually incorporate the results of novel discoveries, target- and immunotherapeutic agents were also introduced into the treatment of melanoma: the BRAF inhibitor vemurafenib was officially registered in 2012, and the CTLA4 inhibitor ipilimumab was accepted in 2011, while the PD1 inhibitors nivolumab and pembrolizumab were accepted in 2015. Our working group has already published experiences about the application of the CTLA4 inhibitory strategy in melanoma [8]. However, Hungarian population-based results with anti-PD1 are only available on a limited basis.

The purpose of the recent study was systematically analyzing the melanoma patient pool at the Department of Dermato-oncology in the Hungarian National Institute of Oncology, focusing on advanced-stage cases that received nivolumab or pembrolizumab therapy. Through our material, we aimed to provide a comprehensive picture about efficacy and the concomitant autoimmune complications of the anti-PD1 strategy and assess their potential association.

## 2. Materials and Methods

### 2.1. Patients

During our retrospective, single-institute analysis, 222 advanced melanoma patients were selected that were treated with anti-PD1 (pembrolizumab, nivolumab) therapy between March 2015 and December 2020. Immunotherapy was applied according to the decision of a multidisciplinary oncology team at the dosage of the approved prescription information. Inclusion criteria were as follows: at least 18 years of age; unresectable or metastatic melanoma; ECOG 0, 1 or 2 performance status; first or higher therapeutic lines; and in cases of cerebral metastasis, the absence of neurological symptoms. Patients suffering from systemic autoimmune diseases and under steroid therapy of more than 8 mg/day methylprednisolone were excluded. Due to funding limitations, BRAF-positive patients could only receive treatment as a second line of therapy after inefficacy of the targeted therapy occurred.

Response was evaluated by computed tomography and magnetic resonance imaging of the brain at weeks 12, 16, and every 3 months thereafter according to irRC (immune related Response Criteria) [9]. The Adverse Events were monitored at each visit based on the CTCAE (Common Terminology Criteria for Adverse Events) version 4.03. In cases of intolerable grade II or grade III–IV adverse events, dose omission or discontinuation was recommended. Physical examination and laboratory tests at each visit were performed. The following predictive parameters were recorded: gender, age, ECOG (Eastern Cooperative Oncology Group) performance status, stage based on AJCC 8th ed. (American Joint Committee on Cancer) [10], BRAF mutation status of primary tumor or metastasis, LDH level at enrollment, autoimmune and other drug-related side effects.

### 2.2. Statistical Analysis

Next to descriptive statistics, survival analysis was performed. Overall survival (OS) and progression-free survival (PFS) were evaluated using the Kaplan–Meier method and log-rank statistics. Univariate and multivariate analyses of predictive factors were assessed by using Cox’s regression model. Differences were considered statistically significant when the *p*-value proved to be under 0.05. All statistical calculations were performed by Statistica 13.4 (TIBCO Software, Palo Alto, CA, USA).

### 2.3. Ethical Permission

The study was conducted under the ethical permission of the Scientific and Research Ethics Committee of the Medical Research Council (approval number: BMEÜ/385-1/2022EKU) and was carried out in accordance with the Code of Ethics of the World Medical Association (Declaration of Helsinki) for experiments involving humans.

## 3. Results

### 3.1. General Characteristic of the Studied Melanoma Patient Group Treated by Checkpoint Inhibitors

Based on the decision of a multidisciplinary oncology team, 222 patients suffering from advanced melanoma were treated with anti-PD1 monotherapy (pembrolizumab, nivolumab) outside of clinical trial, and 142 men (63.9%) and 80 women (36.1%) received the treatment. The median age of patients was 67 years, ranging from 27 to 90. Out of the patients, 183 (82.4%) were in ECOG 0 status, 133 (14.9%) in ECOG 1, and 6 (2.7%) in ECOG 2. From the whole exploratory group 173 patients received pembrolizumab (77.9%), while 49 received nivolumab (22.1%) therapy. Anti-PD1 agents were applied as the first-line therapy for 150 patients (67.6%), while 72 patients (32.4%) received treatment in the second or third lines of therapy.

According to the 8th version of AJCC, 15 patients had unresectable stage III melanomas (6.8%), 21 were in M1a stage (9.5%), 62 were in M1b stage (27.9%), 89 were in M1c stage (40.0%), and 35 patients were in M1d stage (15.8%) [10]; 30 patients had brain metastasis at the start of therapy (13.5%). The LDH level was in the normal range in 144 cases (64.9%) and above normal level in 78 cases (35.1%). BRAF mutation was detected in 50 tumors (22.5%). Efficacy and safety were evaluated using the data available in May 2021, at which point 106 (47.7%) patients were alive, and 116 (52.3%) had died. The median follow-up time was 16 months (range: 0–64), and the therapy’s median duration was 7 months (range: 0–60) (Table 1).

In total, 96 patients had confirmed objective response (43.3%), out of which 25 cases had CR (11.3%) and 71 (32.0%) had PR; 37 patients showed stable disease (16.7%). PD appeared in 73 patients (32.9%), and in the case of 16 patients, therapy response could not be evaluated (7.2%).

**Figure 1:**
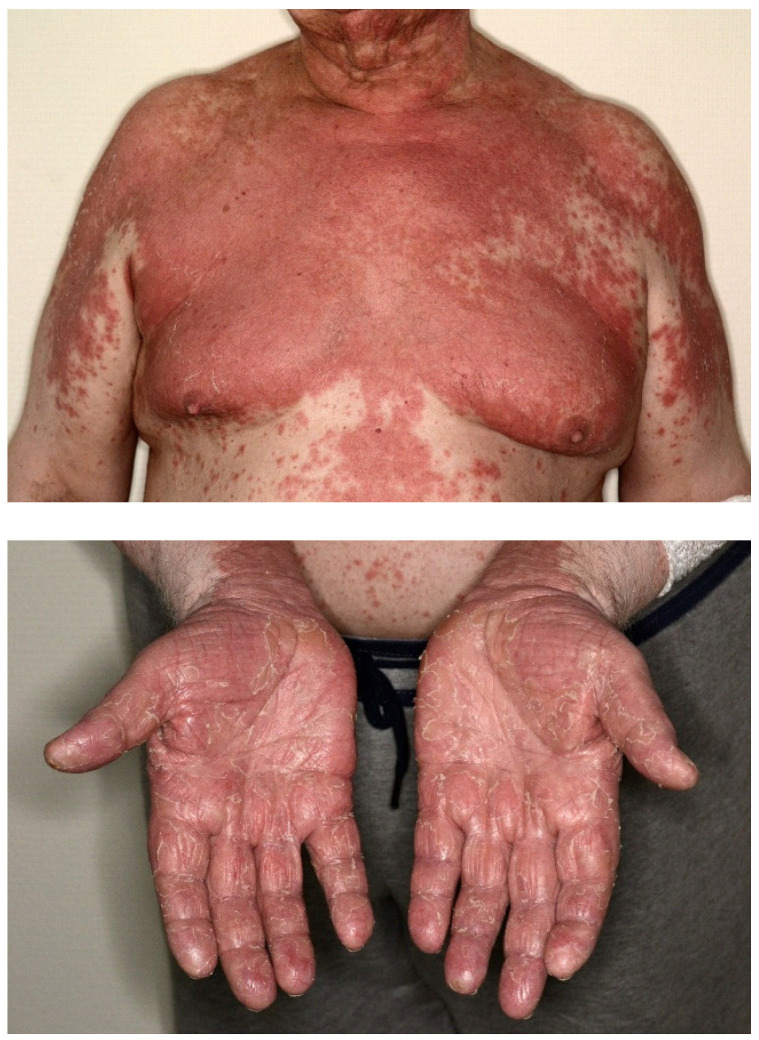
Histologically confirmed PD-1 inhibitor induced pityriasis rubra pilaris (from the photo archives of the Hungarian National Institute of Oncology).

Drug-related adverse events were detected in 124 patients (55.9%). Autoimmune side effects were found in 79 patients (35.5%); the most frequent form was hypothyroidism in 22 (9.91%), followed by vitiligo in 18 (8.12%), other skin symptoms in 13 (5.86%), and pneumonitis in 9 patients (4.05%) (Table 2, Figure 1).

Autoimmune side effects were categorized as grade 1 in 25 (11.26%), grade 2 in 37 (16.67%), and grade 3 in 14 (6.31%) cases; 11 side effects (which required no intervention) were not categorized. One patient (0.45%) died due to fulminant pneumonitis. In the case of 19 patients (8.56%), we ceased the therapy due to autoimmune side effects. Other, not immune-related adverse events occurred in 45 cases (20.3%), most commonly elevated liver function and lipase values.

### 3.2. Progression-Free Survival of the Studied Melanoma Patient Group Receiving Checkpoint Inhibitor Therapy

**Figure 2:**
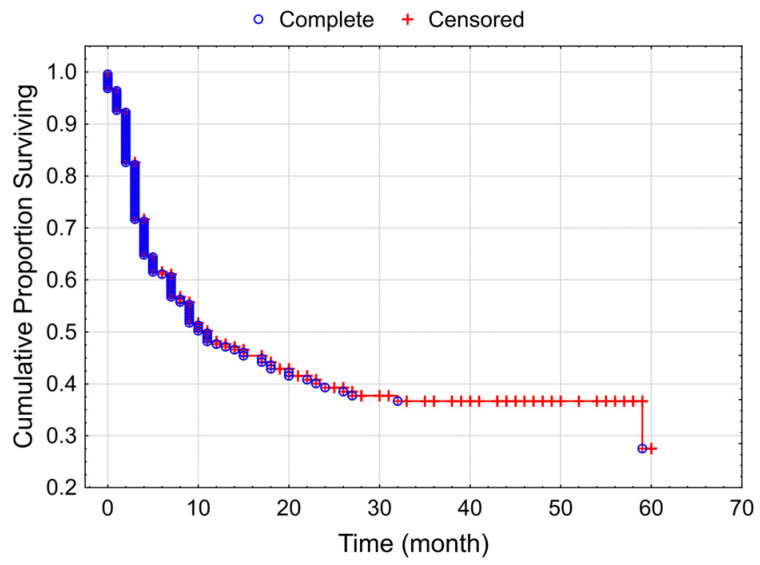
Kaplan–Meier curve for progressive-free survival of the total studied patient population with melanoma (median PFS: 10 months).

The median PFS of the whole study population was 10 months (range: 0 to 60 months) (Figure 2).

Age, gender, ECOG status of the patients, and the type of anti-PD1 therapy did not show significant correlation with the PFS, nor with the stage of the disease. We have not found any statistically significant correlation between brain metastases and PFS either, which could be explained by selection criteria for stable cerebral metastasis. We have not found any relationship between BRAF mutation and progression-free survival.

**Figure 3:**
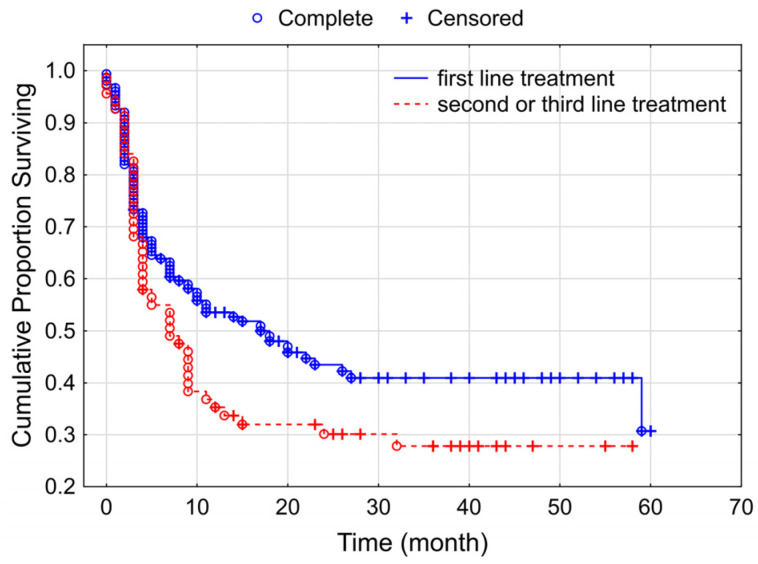
Kaplan–Meier curves for progressive-free survival according to the therapeutic line of anti-PD1 therapy groups among our studied melanoma group of patients (*p* = 0.056).

The comparison of therapeutic-line level revealed an almost significant difference (*p* = 0.056) with the PFS value: median survival was 17 months in the case of first-line therapy, while it was 7 months for second- and third-line therapies (Figure 3).

**Figure 4:**
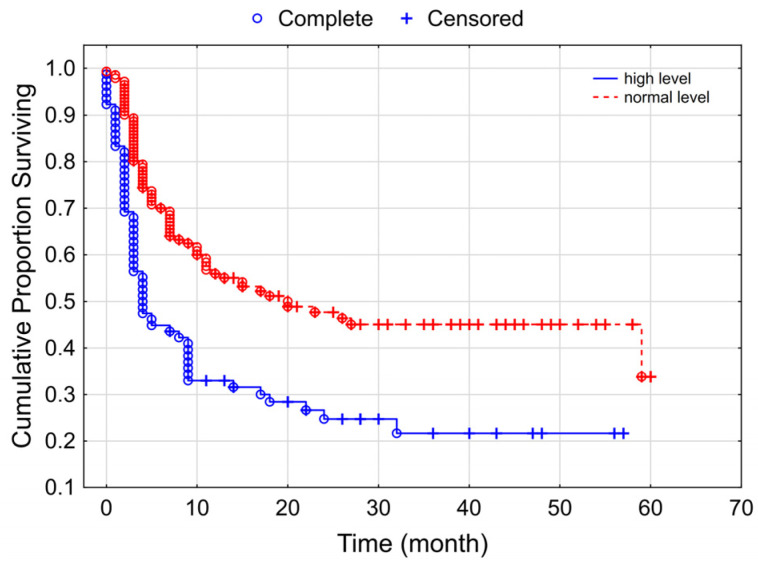
Kaplan–Meier curves for progressive-free survival according to the initial LDH values (*p* = 0.00011).

A significant correlation between the baseline LDH value and PFS was also found: in the case of LDH values above the normal range, median PFS was 4 months, whereas in the case of LDH values within the normal range, it was 20 months (Figure 4).

**Figure 5:**
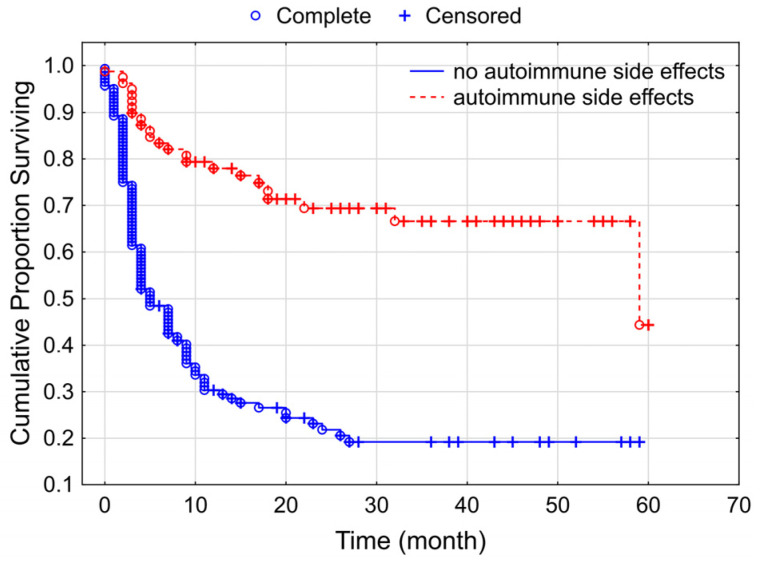
Kaplan–Meier curves for progressive-free survival according to the appearance autoimmune side effects among our studied group of patients with melanoma (*p* < 0.000001).

The below Kaplan–Meier curve demonstrated a highly significant correlation between autoimmune side effects and progression-free survival: regardless of the severity, autoimmune side effects associated with longer progression-free survival. The median survival of patients without autoimmune side effects was 5 months, as opposed to 59 months in the case of those with autoimmune side effects (Figure 5).

Analyzing the parameters in a multivariate analysis, we concluded that in accordance with the Kaplan–Meier analysis, the initial LDH value (*p* = 0.003) and the autoimmune side effects (*p* < 0.000001) proved to be independent predictors of progression-free survival (Table 3).

### 3.3. Overall Survival of the Studied Melanoma Patient Group Treated by Checkpoint Inhibitors

**Figure 6:**
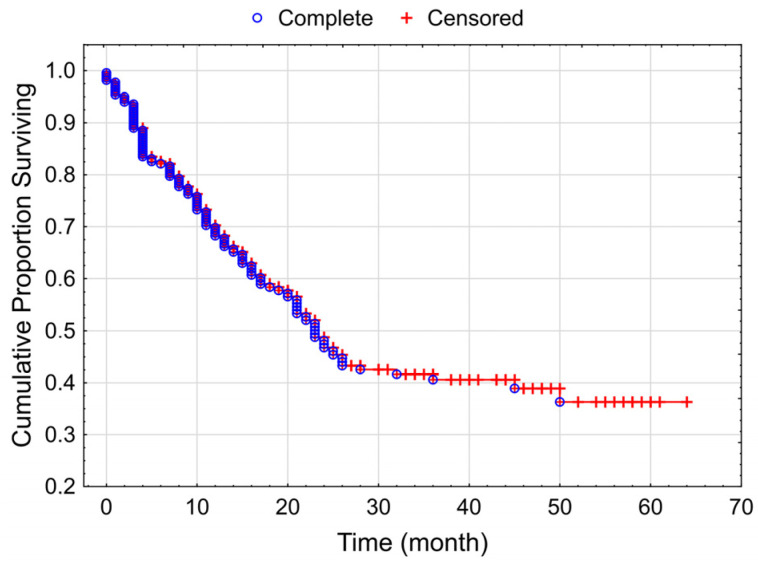
Kaplan–Meier curve for overall survival of the total studied patient population with melanoma (median OS: 23 months).

Median OS proved to be 23 months for the patient population (range: 0–64 months) (Figure 6). A statistically highly significant relationship between the baseline LDH value and OS (*p* = 0.00008) was found. In the case of normal LDH values, median survival was 28 months, and above the normal levels overall it was 14 months.

**Figure 7:**
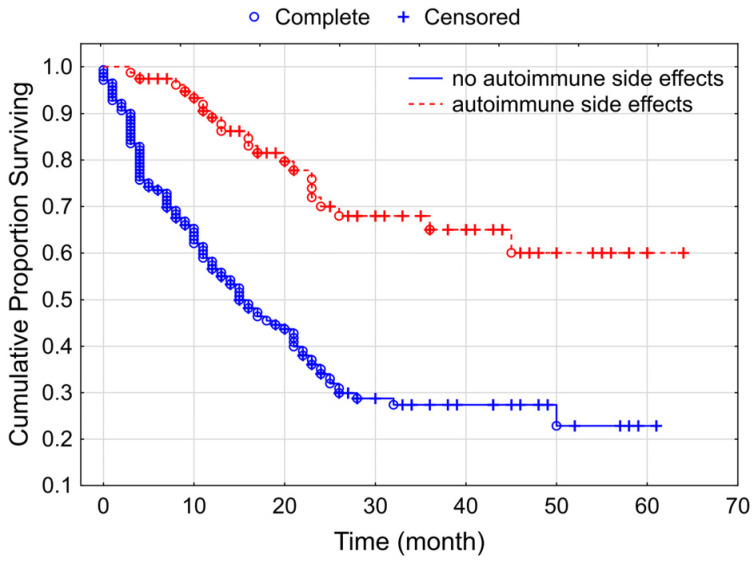
Kaplan–Meier curves for overall survival according to the appearance autoimmune side effects among our studied group of patients with melanoma (*p* < 0.000001).

The survival difference was also significant between the autoimmune side effects group and the group that lacked them, to the benefit of the former (*p* < 0.000001). In the favorable survival group, we did not reached the median value, while in the other category, median survival was 15 months (Figure 7).

**Figure 8:**
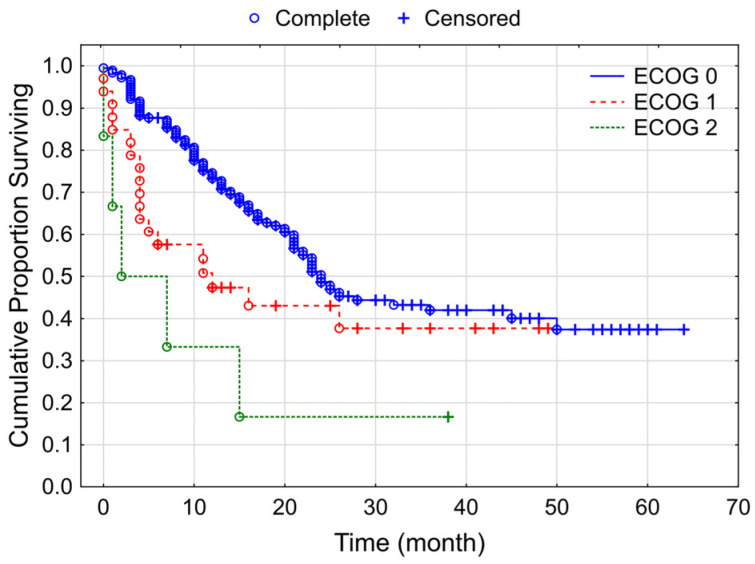
Kaplan–Meier curves for overall survival according to ECOG status among our studied group of patients with melanoma (*p* = 0.001).

In terms of overall survival, ECOG status showed a significant correlation; it was 24 months in the ECOG 0 group, in the ECOG 1 it was 12 months, while in the ECOG 2 group, median survival was a mere 2 months (*p* = 0.001) (Figure 8).

We have not found any correlation between the patients’ age, gender, the type of therapy, the stage of the disease, the presence or absence of brain metastasis, BRAF mutations, and survival using Kaplan–Meier analysis.

The independent parameters correlated with OS were autoimmune side effects (*p* < 0.000001; RR 3.547; 95%CI (2.183–5.762)), the baseline LDH value (*p* = 0.005; RR 0.55; 95%CI (0.362–0.836)), the ECOG status (*p* = 0.013; RR 0.327; 95%CI (0.127–0.843)), and the gender of the patient (*p* = 0.024; RR 0.633; 95%CI (0.425–0.942)) (Table 4).

## 4. Discussion

The poor outcome of advanced melanoma had not change for decades, and only the introduction of target therapy and immunotherapy have opened a new horizon in the management of these patients. Even though the immunology of melanoma had been studied for a long time, the discovery of checkpoint inhibitors meant a true breakthrough [11]. Anti-CTLA4 and anti-PD1 therapies and their combined usage have resulted in an effective tumor response by reactivating T-lymphocytes. In BRAF-positive melanoma, Anti-PD-L1 atezolizumab in combination with the BRAF-MEK inhibitor vemurafenib + cobimetinib provided a survival advantage compared to placebo with the same combination of target therapy [12]. The most recently accepted immune modulation agent inhibits LAG-3 (lymphocyte-activation-gene-3), which, in combination with nivolumab, provided a further therapeutic response in melanoma [13].

From 2015 onward, pembrolizumab and nivolumab monotherapy have been applied among 222 patients in our department. Therapeutic response was 43%, which proved to be much more favorable than that of our ipilimumab-treated patients (17%). During the recent study, our advanced melanoma patient group showed 10 months of median PFS and 23 months of median OS. These values exceeded the outcome of ipilimumab-treated patients (2.7 months and 9.8 months, respectively) that were previously treated in our institute [8]. The KEYNOTE-001 study’s enrolled 665 patients were admitted to pembrolizumab therapy, and survival parameters were very similar to the result of our own study: PFS was 8.3 months and OS was 23.8 months [14]. In the CheckMate-067 study, on the nivolumab arm, the PFS was 6.9 months, while OS proved to be 36.9 months [6].

However, clinical trials provide relatively sterile circumstances, and those results are not always approved from real-life patient samples. Since, in the literature, the majority of the available data demonstrated the results of clinical trials, the outcomes of melanoma patients that were treated in “routine” healthcare systems may add valuable details to assess the efficacy of immunotherapy. The clinical work of Cybulska-Stopa et al. enrolled 1037 patients with metastatic melanoma; 44% of the patients were treated with pembrolizumab and 56% with nivolumab [15]. They found more prolonged overall and progression-free survival among the nivolumab group; however, the difference was not statistically significant. Nevertheless, PFS and OS values of more favorable nivolumab were in the same range as ours (7.5 and 20.0 months, respectively).

The age, gender, ECOG status, stage of disease, presence of a BRAF mutation, and type of the applied anti-PD-1 therapy in our real-world study showed no significant correlation with the PFS value as it was found in the previous published randomized studies [16,17]. We have not found a statistically significant correlation between the presence of brain metastases and PFS either; however, the majority of our patients were symptom-free. There are limited data on the efficacy of anti-PD1 therapy in melanoma patients with brain metastasis; nevertheless, according to previous studies, patients with symptomatic brain metastasis have less favorable outcomes than those without symptoms [18]. Note that our selection criteria indicated that only those patients could be included where clinical symptoms of cerebral metastasis were not present. Similar to the results of other experiences, the level of the therapeutic line showed a nearly significant relationship with the PFS value, since first-line treatment with checkpoint inhibitors was associated with better outcome than those of later applications [19].

Next to known clinico-pathology factors, numerous other biological processes may affect the expected outcome of immunotherapeutic agents in melanoma patients. The immunosuppressive effect of melanogenesis by itself can lead to melanoma progression and resistance to immunotherapy through the activation of glycolysis and hypoxia-inducible factor 1-alpha [20]. In addition, advanced melanoma may produce several neuroregulatory factors and corticosteroids, which protect the tumor from the host responses and also result in resistance against immunotherapeutic agents [21]. However, the recent study focused only on the clinically available prognostic factors, and analysis of these parameters may exceed the frame of our work.

Autoimmune drug-related adverse events developed in 35.6% of our patients. Grade 3 autoimmune side effects occurred in 6.3% of the cases, and grade 5 occurred in the case of 1 patient (0.25%), who died due to fulminant pneumonitis. The most common occurrences were hypothyroidism (9.9%), vitiligo (8.1%), rash (5.9%), and pneumonitis (4.1%). We have found significant (*p* < 0.000001) correlation between autoimmune side effects and PFS. Median PFS was 5 months without autoimmune side effects, while with them it was 59 months. The correlation found was recorded in the literature as well [9,22,23]. In contrast to the results of Suo et al., where only grade 3 or higher-level drug related autoimmune adverse events were associated with more favorable survival rates; in our patients, the more favorable outcome proved to be independent from the severity of autoimmune side effects [19].

Our results are corroborated with Eggermont’s two-arm study of 1011 stage III patients who were randomized for pembrolizumab or placebo; autoimmune side effects were described in 37.4% of the cases with pembrolizumab treatment (in our patients it was 35.6%), and significant correlation was detected between autoimmune side effects and relapse-free survival (*p* = 0.03) [23].

The other significant parameter which correlated with PFS was LDH value; high levesl proved to be a very important negative predictive factor of melanoma in both targeted and immunotherapy [24,25]. In contrast, a previously published study showed more favorable outcome in the case of high baseline LDH levels, where PD-L1/IDO (indoleamine 2,3-dioxygenase) peptide vaccines were used to target PD-L1 and IDO-expressing cells. Both therapy responses (82% vs. 79%), as well as median PFS (30.9 months vs. 19.3 months), proved to be better among the high-LDH level group; however, the limitation of the study was that only 30 patients were enrolled [26]. Our multivariate analysis revealed that the initial lower LDH value (*p* = 0.003) and occurrence of autoimmune side effects (*p* < 0.000001) proved to be independent positive predictors of progression-free survival. Autoimmune side effects (*p* < 0.000001), the LDH value (*p* = 0.005), and the ECOG status (*p* = 0.013), as well as the patient’s gender (*p* = 0.024), were independent predictive factors of OS.

The analysis among a real-life clinical setting showed that the survival rates of melanoma patients treated with anti-PD1 reached that of clinical trials despite the fact that about one-third of our patients received treatment in the second or third line [8]. The efficacy of the two applied different agents (pembrolizumab and nivolumab) were the same.

## 5. Conclusions

We concluded that in the case of melanoma, the range of available immunotherapeutic medicines has widened significantly by now, and anti-PD1 monotherapy still remains an important, effective and safe method. In addition, the expected outcome is more favorable in the event of immune-related side effects.

## Figures and Tables

**Table 1:** Anti-PD1 therapy patient parameters.

Patients	No (%)
**Total**	222 (100%)
**Age (years) (median, min–max)**	67 (27–90)
**Follow-up time (in months) (median, min–max)**	16 (0–64)
**Duration of therapy (in months) (median, min–max)**	7 (0–60)
**Gender**	
Male	142 (63.9%)
Female	80 (36.1%)
**ECOG status**	
0	183 (82.4%)
1	33 (14.9%)
2	6 (2.7%)
**Therapy**	
Pembrolizumab	173 (77.9%)
Nivolumab	49 (22.1%)
**Line of treatment**	
First	150 (67.6%)
Second or third	72 (32.4%)
**Stage**	
Unresectable III	15 (6.8%)
M1a	21 (9.5%)
M1b	62 (27.9%)
M1c	89 (40.0%)
M1d	35 (15.8%)
**Brain metastasis**	
No	192 (86.5%)
Yes	30 (13.5%)
**LDH**	
Normal value	144 (64.9%)
Above normal level	78 (35.1%)
**BRAF mutation**	
Negative	172 (77.5%)
Positive	50 (22.5%)
**Therapeutic response**	
Complete response	25 (11.3%)
Partial response	71 (32%)
Stable disease	37 (16.7%)
Progressive disease	73 (32.9%)
Not available	16 (7.2%)
**Side effect**	
Autoimmune	79 (35.6%)
Other	45 (20.3%)

**Table 2:** The immune-related symptoms of our patient group with melanoma side effects (anti-PD1 therapy patient parameters).

Side Effect	No (%)
Hypothyroidism	22 (9.91%)
Vitiligo	18 (8.12%)
Skin symptoms	13 (5.86%)
Pneumonitis	9 (4.05%)
Colitis	7 (3.15%)
Arthralgia, artritis	5 (2.25%)
Hypophysitis	3 (1.35%)
Pancreatitis	3 (1.35%)
Ocular inflammation	2 (0.90%)
Hyperthyreosis	2 (0.90%)
Drug-induced diabetes	1 (0.45%)
Hemolytic anemia	1 (0.45%)
Systemic autoimmune disease	1 (0.45%)

**Table 3:** Multivariate analysis for determining the correlation between progression-free survival and various predictive parameters among our melanoma patient group.

Parameters	RR ^1^ (95% CI)	*p*
Age	0.995 (0.979–1.011)	0.543
Gender (reference: female)	0.779 (0.532–1.141)	0.2
Line of therapy (reference: second or later)	0.722 (0.425–1.228)	0.229
ECOG (reference: 2)		
0	0.786 (0.282–2.191)	0.445
1	0.976 (0.328–2.908)	0.778
LDH (reference: high)	0.553 (0.377–0.813)	0.003
BRAF (reference: negative)	1.135 (0.597–2.159)	0.7
M-stage (reference: N)		
M1a	1.068 (0.402–2.84)	0.737
M1b	1.867 (0.828–4.208)	0.228
M1c	1.848 (0.858–3.98)	0.153
M1d	0.935 (0.367–2.383)	0.957
Autoimmune side effects (reference: yes)	4.269 (2.685–6.788)	<0.000001

^1^ RR: relative risk.

**Table 4:** Multivariate analysis for determining the correlation between overall survival and various predictive parameters among our melanoma patient group.

Parameters	RR ^1^ (95% CI)	*p*
Age	1 (0.984–1.018)	0.958
Gender (reference: female)	0.633 (0.425–0.942)	0.024
Line of therapy (reference: second or later)	0.632 (0.378–1.057)	0.081
ECOG (reference: 2)	0.327 (0.127–0.843)	
0	0.453 (0.163–1.259)	0.013
1	0.55 (0.362–0.836)	0.487
LDH (reference: high)	1.146 (0.606–2.167)	0.005
BRAF (reference: negative)	2.21 (0.791–6.174)	0.676
M-stage (reference: N)	2.205 (0.909–5.35)	
M1a	2.451 (1.079–5.566)	0.322
M1b	1.779 (0.639–4.952)	0.694
M1c	3.547 (2.183–5.762)	0.259
M1d	1 (0.984–1.018)	0.576
Autoimmune side effects (reference: yes)	0.633 (0.425–0.942)	<0.000001

^1^ RR: relative risk.

## Data Availability

Under special request, the authors will share anonymized datasets.
